# Small bowel volvulus secondary to torsion of a mature cystic teratoma in the first trimester of pregnancy: A case report

**DOI:** 10.1016/j.crwh.2025.e00776

**Published:** 2025-12-24

**Authors:** Wondwosen Mengist Dereje, Biniam Gezahagn zewude, Gashaw Tesfa Aragaw, Meredin Nuru Seyide, Asmare Getaneh Aynishet, Misganaw Abere Worku, Samuel Addisu Abera, Gebremariam Maru Yemru, Alem Demissie Bogale, Asratu Getnet Amare, Fasil Tadesse Ashagrie

**Affiliations:** a University of Gondar, College of Medicine and Health sciences, Gondar 196, Ethiopia; b University of Gondar, College of Medicine and Health sciences, Department of Surgery, Gondar 196, Ethiopia; c University of Gondar, College of Medicine and Health sciences, Department of Gynecology and obstetrics, Gondar 196, Ethiopia; d University of Gondar, College of Medicine and Health sciences, Department of Pathology, Gondar 196, Ethiopia

**Keywords:** Small bowel volvulus, Pregnancy, Cyst torsion, Laparotomy, Case report

## Abstract

Small bowel volvulus is a rare but potentially life-threatening surgical emergency. Its occurrence in association with ovarian cyst torsion during pregnancy is exceedingly rare, with few cases reported. To the authors' best knowledge, this case represents the first documented instance occurring in the first trimester.

A 37-year-old woman (gravida 3, para 2) at 12 weeks +2 days of gestation was referred with a 16-h history of crampy abdominal pain, initially infraumbilical and later diffuse, accompanied by multiple episodes of vomiting, which progressed from ingested matter to bilious, and 12 h of progressive abdominal distension.

On presentation, she appeared acutely ill and in severe pain, with abdominal examination revealing generalized peritonitis. She was promptly started on intravenous antibiotics and fluid resuscitation, and an emergency laparotomy was performed. Intraoperatively, a torsioned adnexal mass, gangrenous right ovary, fallopian tube, and a segment of small bowel were identified and resected. The patient left the operating room with stable vital signs and was subsequently transferred to the ward, where she recovered well. Six months later, she delivered a healthy 3.5 kg female infant via repeat cesarean section, with no complications.

Although small bowel volvulus is rare, it must be considered in patients with intestinal obstruction. Delayed recognition can cause ischemia, necrosis, and perforation, so maintaining suspicion, performing timely investigations, and initiating early surgical intervention are essential to improve outcomes and reduce morbidity and mortality.

## Highlights

• Small bowel volvulus is a rare, life-threatening obstruction with high ischemic risk.
• In early pregnancy, it is uncommon and often missed due to overlapping symptoms.
• Primary volvulus is anatomical; secondary results from malrotation, tumors, or cysts.
• CT findings help diagnosis, but peritonitis mandates urgent surgical intervention.
• Early surgery reduces mortality, which is high in advanced gangrenous disease.

## 1 Introduction

Small bowel volvulus (SBV) is rare [1]. Approximately 75 % of all volvulus cases occur in the colon, with the remaining 25 % involving the small bowel. SBV occurs when bowel loops abnormally twist around the axis of their own mesentery, causing rotation and occlusion of the mesenteric vessels. This vascular compromise can lead to intestinal obstruction, venous congestion, gangrene, and eventual perforation. The condition is more frequently observed in neonates and young adults and is exceedingly uncommon in older adults [2].

Pregnancy complicated by intestinal obstruction is very rare, with an incidence ranging from approximately 1 in 5000 to 1 in 66,000 pregnancies [[1], [2], [3]]. Intestinal volvulus accounts for about 25 % of acute bowel obstructions in pregnant women [3]. Although several risk factors have been described, the most commonly reported include a high-fiber diet (often attributed to African origin), chronic constipation, pregnancy (particularly in the third trimester due to displacement and partial compression of the bowel by the gravid uterus), postoperative adhesions, and midgut malrotation [2].

A good outcome depends largely on early diagnosis and prompt surgical intervention [3].

## 2 Case Presentation

A 37-year-old woman (gravida 3, para 2, both children delivered by cesarean section), at 12 weeks +2 days of pregnancy, presented with crampy abdominal pain of 16 h' duration. The pain initially started as mild and infraumbilical but later became severe and diffuse, involving all abdominal quadrants to the point that she was unable to move. She also reported multiple episodes of vomiting during the same period, which initially consisted of ingested food but later became bilious. Four hours after the onset of pain and vomiting, she developed progressive abdominal distension. She denied any vaginal bleeding or history of trauma. She was first taken to a nearby primary hospital, but was referred to the tertiary hospital for further investigation and management.

She could not recall the exact date of her last normal menstrual period but reported being amenorrheic for three months. The pregnancy had been diagnosed two months prior to her current presentation, and early ultrasound estimated the gestational age at 12 weeks and 4 days.

Upon presentation at the tertiary hospital, she appeared acutely ill and in severe pain. Her vital signs were blood pressure 100/60 mmHg, pulse rate 90 beats per minute, respiratory rate 19 breaths per minute, temperature 36.6 °C, and oxygen saturation 97 % on room air.

On abdominal examination, there was an old midline surgical scar; her uterus was consistent with a 14-week gestation. She exhibited diffuse tenderness with rigidity and guarding across all abdominal quadrants. Digital rectal examination revealed a smooth, minimal stool in the rectum with no masses or blood on the examining finger.

Considering her clinical presentation and history of previous cesarean section, generalized peritonitis secondary to small bowel obstruction due to postoperative adhesions was suspected.

After establishing an intravenous (IV) line, she was started on normal saline. A nasogastric tube was inserted for gastric decompression, and intravenous antibiotics (ceftriaxone 1 g IV and metronidazole 500 mg IV) were initiated.

While the IV line was secured, laboratory investigations were requested. The results were as follows: CBC, WBC 8.5 × 10^3^/μl, hemoglobin 11.3 g/dl, hematocrit 37.8 %, platelets 187 × 10^3^/μl; liver enzymes, ALT 15 U/l, AST 34 U/l; renal function, BUN 27 mg/dl; serum creatinine 0.34 mg/dl; serum electrolytes, Na^+^ 136.5 mmol/l, K^+^ 3.87 mmol/l, Ca^2+^ 1.9 mmol/l, Cl^−^ 108 mmol/l.

Although bedside ultrasonography should have been performed in the emergency setting given the patient's pregnancy, it was unavailable at the time. In addition, because of generalized peritonitis, referral to the radiology department for imaging was deferred to avoid delaying management, and a decision for immediate intervention was made.

Considering the generalized peritonitis, no further investigations were deemed necessary.

The obstetrics and gynecology team was consulted in the emergency department. The patient was jointly evaluated by the surgical and obstetrics and gynecology teams, and generalized peritonitis secondary to gangrenous bowel was suspected; therefore, an emergency laparotomy was planned.

The case was discussed with the patient and her family, and surgical intervention was offered, to which they agreed. After obtaining informed consent, she was taken to the operating room for a laparotomy, with the obstetrics and gynecology team on standby.

**Fig. 1:**
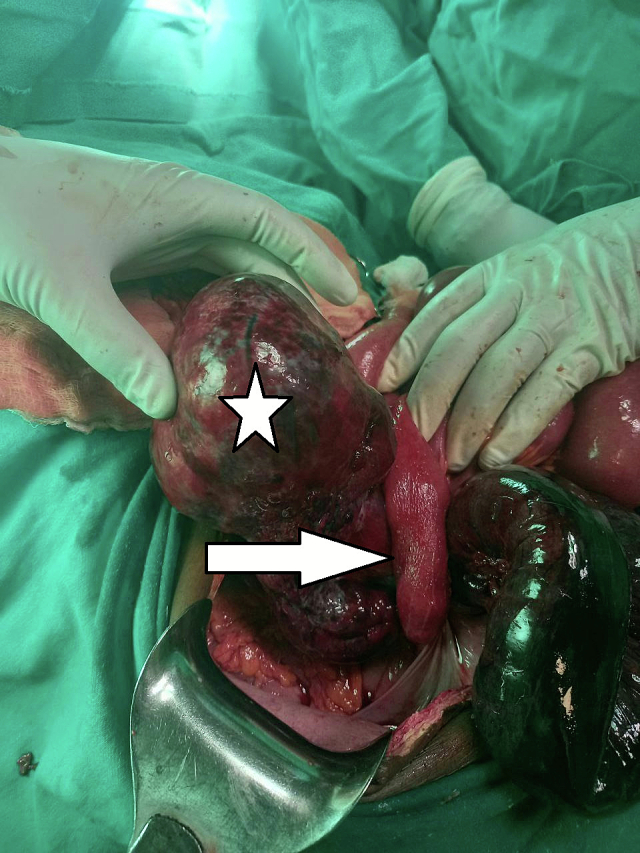
Small bowel segmented rotated 360^0^ (arrow) along the right ovary, right fallopian tube, and large adnexal mass (star).

The patient had old midline surgical scar from her previous deliveries. A midline incision was made and the peritoneal cavity was accessed, revealing approximately 500 ml of hemorrhagic fluid. Approximately 20 cm of small bowel, located 5 cm from the ileocecal valve, was twisted 360° counterclockwise around the cystic mass, the right ovary, and the right fallopian tube (Fig. 1). This segment of bowel was frankly gangrenous, and an additional 10 cm of ileum appeared ischemic.

**Fig. 2:**
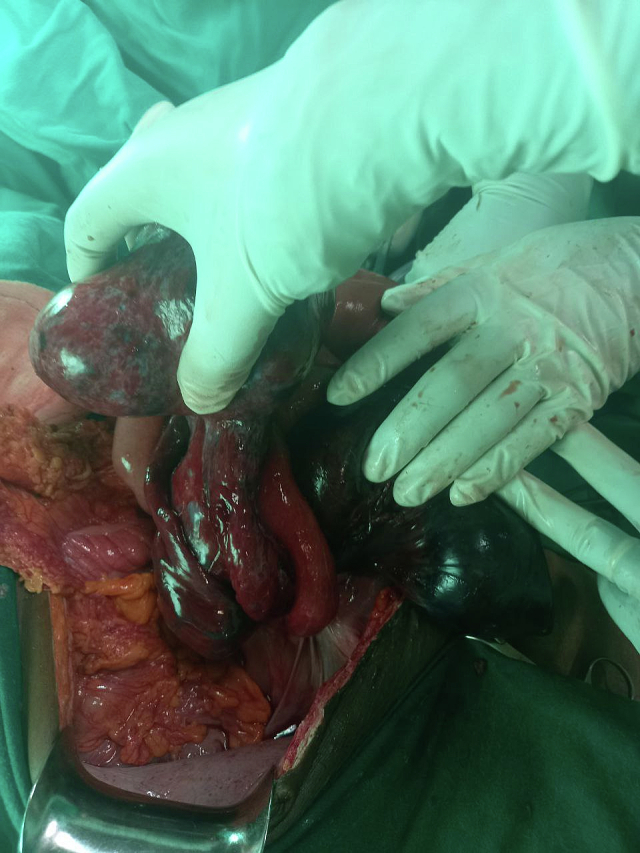
The adnexal mass and the bowel segment entangled over it.

There was also a 12 × 10-cm right adnexal mass involving the right ovary and right fallopian tube. The mass had undergone a 720° clockwise torsion (two full rotations). The mass, right ovary, and right fallopian tube were all gangrenous (Fig. 2). The obstetrics and gynecology team with the surgical team performed the operation collaboratively.

**Fig. 3:**
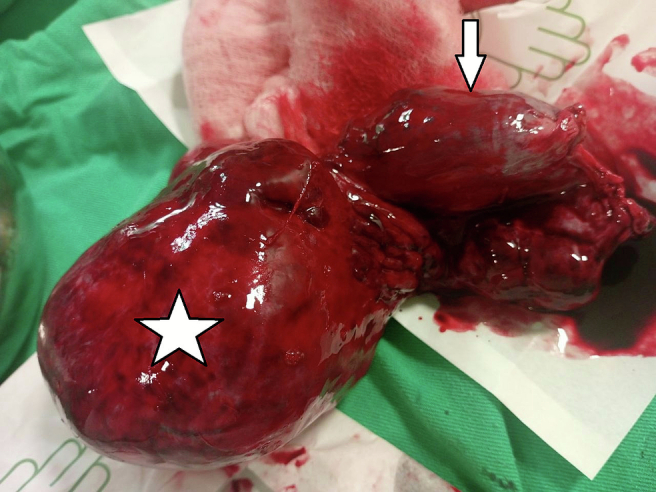
Right adnexectomy; cystic mass (star), right fallopian tube (arrow).

**Fig. 4:**
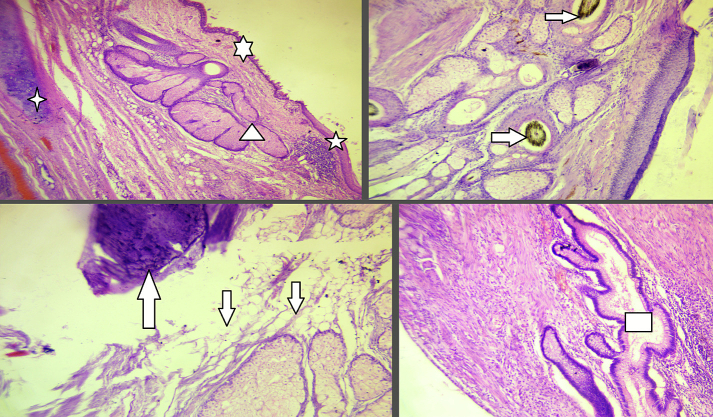
Mature teratoma containing; cartilage (); stratified squamous epithelium (); pseudostratified ciliated columnar (); foci of bone formation (); sebaceous glands (); adipose tissue (); Mucin producing columnar ().

An ileal resection with end-to-end anastomosis was performed, along with a right adnexectomy (Fig. 3). The cystic mass was sent for histopathological evaluation, which confirmed mature cystic teratoma (Fig. 4). The bowel and fallopian tube sample was also sent and it showed normal histology report.

The patient was transferred to the post-anesthesia care unit (PACU) with stable vital signs, where she remained for 12 h before being moved to the recovery area for an additional 24 h. After a total of 36 h in the PACU and recovery room, she was transferred to the surgical ward.

She remained on the ward for five days under close monitoring and was subsequently discharged with follow-up appointments at the outpatient surgical clinic and antenatal care (ANC) clinic. At her two-, four-, and six-week follow-up visits, she reported no new complaints and was discharged from the surgical service. She was then referred to her primary hospital to continue routine ANC visits.

She continued her regular ANC visits at the primary hospital, and considering her two previous cesarean scars, the long distance, and difficulty accessing transportation, she was referred to the general hospital for admission and better management at 37 weeks +1 day. She stayed at the general hospital for one week, and a repeat cesarean section was performed at 38 weeks +2 days. She delivered a healthy 3.5 kg female neonate with no complications.

She remained at the general hospital for three days postpartum and was discharged with advice on exclusive breastfeeding, vaccination, hygiene, and follow-up at the gynecology and obstetrics outpatient clinic. Her outpatient visits at one week and two weeks after discharge from the general hospital were normal, with no complications, and she was subsequently discharged from care.

## 3 Discussion

Acute intestinal obstruction encompasses a range of conditions that present as an acute abdomen, including common clinical scenarios such as hernias (internal or external), ovarian torsion in girls, ingestion of foreign bodies, or midgut volvulus associated with malrotation [4]. Although small bowel volvulus accounts for less than 5 % of all intestinal obstructions, it represents approximately 25 % of small intestinal obstructions [3]. Despite its rarity in clinical practice, it can be life-threatening due to the high risk of strangulation and ischemic necrosis. It occurs more commonly in neonates and young adults and is very rare in older adults [5].

Globally, the incidence of small bowel volvulus ranges from 0.00001 % to 0.19 % [6]. Volvulus is also more commonly observed in children than in adults [7]. The incidence of small bowel volvulus is higher in Middle Eastern, Asian, Central African countries, and Finland compared to Western countries [8]. However, some literature from Asia reports that the incidence of SBV is low in Japan and Taiwan [8]. In Western countries, the incidence of small bowel volvulus is 1.5 to 5.7 per 100,000 adults [6], with 3 to 6 % of these patients presenting with intestinal obstruction [6]. In Africa and the Asian continent, the incidence ranges from 24 to 60 per 100,000 adults [6], and 20 to 50 % of patients present with intestinal obstruction [6].

Small bowel volvulus can be classified into primary and secondary types. Primary small bowel volvulus occurs in a normal abdominal cavity without any underlying anatomical abnormalities or predisposing factors, whereas secondary small bowel volvulus arises in the presence of congenital or acquired predisposing lesions [5,9]. The incidence of primary small bowel volvulus is 48 %, with no identifiable cause [2]. Primary small bowel volvulus is often associated with anatomical features such as an unusually long small intestine, a broad but fat-free mesentery with a narrow base [11], and increased gut motility [15]. Dietary habits, including prolonged fasting, are also considered contributing factors in the development of small bowel volvulus [8].

The sudden passage of a large, bulky meal into the proximal jejunum increases its weight, causing it to shift inferiorly or toward the left lower quadrant due to the minimal resistance within the pelvis. Consequently, the distal jejunum and ileum are forced into a clockwise rotation toward the right lower quadrant, contributing to mesenteric torsion. Young age and a strong muscular abdominal wall further facilitate twisting of the bowel loop [9,15].

Secondary small bowel volvulus is multifactorial, with postoperative adhesions being the most common cause, accounting for 74 % of cases [2,7,8,10]. Other secondary causes of small bowel volvulus include midgut malrotation [2,11], Meckel's diverticulum [2,11], adhesions [12], third-trimester pregnancy [2,11,12], chylolymphatic mesenteric cysts, intestinal duplication, ovarian cysts, meconium pseudocysts, congenital mesenteric defects, persistent omphalocele cysts, simultaneous pancreas and kidney transplantation, and paraduodenal hernias [2]. Small bowel volvulus in association with an ovarian cyst is rare in medical practice.

Diagnosing an acute abdomen during pregnancy is generally challenging due to several factors: (a) the enlarging uterus displaces intra-abdominal organs, making physical examination difficult; (b) nausea, vomiting, and abdominal pain are common symptoms in normal pregnancy, which can mask underlying pathology; and (c) there is a general reluctance to operate unnecessarily on a gravid patient. Appendicitis is the most common cause of acute abdomen during pregnancy (1 in 500–2000 pregnancies), accounting for approximately 25 % of non-obstetric surgical interventions. Acute cholecystitis and bowel obstruction are the second and third most common causes, occurring in 1 in 1600–10,000 and 1 in 1500–16,000 pregnancies, respectively [13,14].

Volvulus is not only rare during pregnancy but also tends to occur most often in the third trimester, when the gravid uterus significantly displaces abdominal structures. In the reported case, the volvulus occurred during the first trimester, making it exceptionally uncommon and therefore noteworthy for publication.

Intestinal obstruction complicating an ovarian cyst is rare and occurs most commonly in neonates (3 %). In studies by Duran A. et al. and Sivaslıoğlu A.A. et al., 19 cases were reported, with only two occurring in adults [14,15]. Intestinal complications typically arise when the ovarian cyst exceeds 10 cm in size, often necessitating emergent surgical intervention [16]. Two mechanisms have been proposed to explain these complications: adhesion-related torsion and the compressive effect of a large cyst on the surrounding bowel [14,15].

Ali Duran et al. reported a case of a giant ovarian cyst compressing the small intestine, ultimately leading to gangrene [14]. Ahmet Akın Sivaslıoğlu et al. described a rare case in which ovarian cyst torsion caused ileal gangrene due to compression and twisting of the mesenteric vessels [15]. The clinical features of small bowel obstruction are often nonspecific, making the diagnosis challenging [16].

The most common presenting feature of small bowel volvulus is abdominal pain [8,10]. The severity of the pain is directly related to the degree of vascular occlusion rather than the extent of intestinal obstruction, and its intensity often does not correlate with clinical findings, as only 26 % of patients present with peritoneal signs [17]. Although peritonitis at presentation is rare, in the reported case, the patient presented with generalized peritonitis, necessitating immediate surgical intervention.

Other clinical features include periumbilical pain unrelieved by analgesics, accompanied by nausea (83 %), vomiting (100 %), abdominal distension (55 %), and signs of peritonism (14–26 %). Fever, tachycardia, and peritonism occur in 90 % of cases with bowel gangrene, necessitating urgent surgical intervention. Hematological findings typically include leukocytosis above 18,000 cells/mm^3^, elevated amylase and lactate levels (55 %), and metabolic acidosis (25 %); however, no laboratory test reliably distinguishes gangrenous from non-gangrenous bowel [18].

Preoperative diagnosis can be challenging and may be further complicated by pregnancy, labor, or the post-cesarean section state. It has been proposed that the gradual enlargement of the uterus during pregnancy can cause partial small bowel obstruction, resulting in proximal distension and torsion at points of fixation [19]. Similarly, the marked increase in uterine size during the third trimester—and its sudden reduction during the puerperium—may predispose patients to small bowel volvulus [2,11]. During these periods, maintaining a high index of suspicion for volvulus is essential for timely diagnosis.

Plain abdominal radiography can demonstrate nonspecific signs of small bowel obstruction, such as dilated small bowel loops with air-fluid levels and, occasionally, pneumatosis intestinalis or thumbprinting, which are indicative of intestinal ischemia [20]. Ultrasonography is sensitive in infants with small bowel obstruction due to small bowel volvulus, but its utility in adults is limited because it is operator-dependent. When successful, ultrasonography may reveal either a whirlpool sign or the classic barber pole sign [12]. Parcos et al. were the first to describe the whirlpool sign on ultrasonography and reported a sensitivity of 89–100 % in diagnosing small bowel volvulus. They also noted that the absence of a whirlpool sign does not rule out small bowel volvulus [2].

CT scan is the investigation of choice for small bowel volvulus, with a sensitivity of 60–100 % and specificity of 90–95 % [9,16]. The characteristic feature on CT is the typical whirl pattern formed by the bowel wrapping around the superior mesenteric artery (SMA) [9]. However, in patients presenting with small bowel volvulus complicated by bowel perforation and generalized peritonitis, imaging should be avoided, as it may delay urgent surgical intervention. Such patients require immediate laparotomy to prevent progression to sepsis and septic shock. In the reported case, the patient presented with generalized peritonitis, and therefore no imaging was performed.

Small bowel volvulus is a surgical emergency that requires prompt surgical intervention to prevent serious complications [20]. It is important to maintain physician awareness of this rare condition, in order to ensure accurate preoperative evaluation and provide timely, definitive surgical treatment [20].

Once small bowel volvulus is suspected, there is no role for conservative treatment, and immediate laparotomy is indicated, as volvulus with compromised vascular status carries a high risk of gangrene [13]. In pregnant women presenting with suspected small bowel volvulus, a multidisciplinary approach involving both surgeons and obstetricians is crucial, as demonstrated in the reported case. Fortunately, small bowel perforation is uncommon, and in cases of necrosis, resection with primary anastomosis is generally safe [19]. In the reported case, the patient presented with gangrenous small bowel as well as a gangrenous cystic mass involving the right ovary and right fallopian tube, adding further complexity to the management.

Mortality in non-infarcted small bowel volvulus ranges from 5 % to 35 % [2,5,8] and can reach up to 100 % when associated with necrotic bowel [5,11]. In the reported case, although the patient presented late with complications, immediate surgical intervention prevented progression to sepsis and septic shock, leading to a favorable outcome.

## 4 Conclusion

Small bowel volvulus is a rare but potentially life-threatening cause of intestinal obstruction, with outcomes heavily dependent on early diagnosis and prompt surgical intervention. Although it more commonly affects neonates and young adults, it can occur in unusual settings, including pregnancy and in association with ovarian cysts, as demonstrated in this case. Imaging studies, particularly CT and Doppler ultrasound, aid in diagnosis, but in cases of generalized peritonitis, immediate surgical exploration is essential to prevent sepsis and bowel necrosis. Timely laparotomy or laparoscopic intervention can significantly improve outcomes, even in complex cases with gangrenous bowel and associated gynecological pathology.

## Contributors

Wondwosen Mengist Dereje contributed to conception of the case report, acquiring and interpreting the data, drafting the manuscript, undertaking the literature review and revising the article critically for important intellectual content.

Biniam Gezahagn zewude contributed to patient care and interpreting the data.

Gashaw Tesfa Aragaw contributed to patient care and interpreting the data.

Meredin Nuru Seyide contributed to patient care and interpreting the data.

Asmare Getaneh Aynishet contributed to patient care and interpreting the data.

Misganaw Abere Worku contributed to conception of the case report and acquiring the data.

Samuel Addisu Abera contributed to conception of the case report and acquiring the data.

Gebremariam Maru Yemru contributed to conception of the case report and acquiring the data.

Alem Demissie Bogale contributed to conception of the case report and acquiring the data.

Asratu Getnet Amare contributed to conception of the case report and acquiring the data.

Fasil Tadesse Ashagrie contributed to patient care, conception of the case report, and acquiring and interpreting the data.

All authors approved the final submitted manuscript.

## Patient consent

Written informed consent was obtained from the patient for the publication of the case report and accompanying images.

## Provenance and peer review

This article was not commissioned and was peer reviewed.

## Funding

No funding from an external source supported the publication of this case report.

## Declaration of competing interest

The authors declare that they have no competing interest regarding the publication of this case report.
